# Analysis of the efficacy of autologous peripheral blood stem cell transplantation in high-risk neuroblastoma

**DOI:** 10.7150/ijms.76305

**Published:** 2022-09-25

**Authors:** Jin Yan, Li Jie, Yang Jiaxing, Cao Yanna, Li Zhanglin, Li Zhongyuan, Wang Daowei, Zhao Guangzong, Zhong Benfu, Yan Jie, Zhao Qiang

**Affiliations:** Department of Pediatric Oncology, Tianjin Medical University Cancer Institute & Hospital, National Clinical Research Center for Cancer, Key Laboratory of Cancer Prevention & Therapy of Tianjin, Tianjin's Clinical Research Center for Cancer, Tianjin 300060, China.

**Keywords:** Neuroblastoma, High-risk, Stem cell transplant, Children, Treatment

## Abstract

**Objective:** This study aimed to analyze the efficacy of autologous peripheral blood stem cell transplantation for high-risk neuroblastoma in China.

**Methods:** The data of 90 high-risk neuroblastoma patients treated with the CCCG-NB 2015 regimen were reviewed. The baseline clinicopathological characteristics and prognosis were analyzed and compared. In addition, the prognoses of tandem autologous stem cell transplantation and single autologous stem cell transplantation groups were compared.

**Results:** The results of survival analysis showed that autologous peripheral blood stem cell transplantation based on this pretreatment regimen significantly improved the prognosis of children in the high-risk group. The 3-year event-free survival (EFS) and overall survival (OS) rates for the transplantation group and the nontransplantation group were 65.5% vs. 41.3% (*p*=0.023) and 77.1% vs. 57.9% (*p*=0.03), respectively. There was no difference in the distribution of baseline clinical case characteristics between the single transplantation group and the tandem transplantation group (*p*>0.05), and there was no significant difference in EFS and OS between the two groups (*p*>0.05).

**Conclusion:** Based on this pretreatment programme, autologous peripheral blood stem cell transplantation is safe and tolerable and significantly improves the prognosis of children in the high-risk group. The value of tandem autologous stem cell transplantation is worthy of further discussion, which should consider various aspects such as the transplantation medication regimen and the patient's state.

## Introduction

Neuroblastoma originates from the adrenal medulla or the paravertebral sympathetic nervous system and is the most common extracranial solid tumor in children. With the advancement of the risk classification algorithm and tailored treatment, the prognosis has been remarkably improved 1, 2. However, the long-term survival of children assigned to the high-risk group is still poor, even under a comprehensive treatment regimen including intensive induction chemotherapy, surgery, radiotherapy, myeloablative chemotherapy with autologous stem cell transplant, and immunotherapy 3-5.

Autologous peripheral blood stem cell transplantation, which attempts to eradicate minimal residual disease with myeloablative chemotherapy and rescue by autologous stem cells (collected from peripheral blood in the previous treatment procedure) to restore bone marrow function, has been included in the standard treatment regimen of high-risk neuroblastoma. A series of studies on autologous peripheral blood stem cell transplantation have been published, however, these have rarely been conducted in China 6. Since 2010, our centre is the first in China to lead the use of tandem autologous stem cell transplantation for the treatment of high-risk neuroblastoma, the related procedures and conditioning protocols for stem cell collection, storage and reinfusion have been developed and incorporated into the high-risk neuroblastoma standard treatment regimen, known as the Chinese Children's Cancer Group (CCCG) Neuroblastoma (NB)-2015 regimen. This study, based on 10 years of clinical experience in our centre, retrospectively analyzed the data of high-risk neuroblastomas treated by the CCCG-NB-2015 regimen and summarized the efficacy and safety of autologous peripheral blood stem cell transplantation, including single transplantation and tandem transplantation. This tandem protocol has not yet been reported, and this is the first report of neuroblastoma stem cell transplantation analysis with a large number of cases in China.

## Materials and methods

### Patient cohort

**Figure 1:**
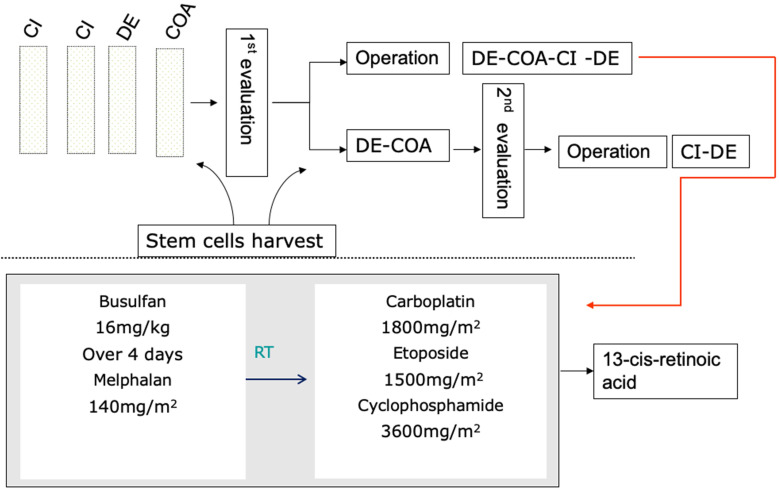
The CCCG-NB-2015 protocol of high-risk neuroblastoma.

A total of 90 children with high-risk neuroblastoma who were admitted to Tianjin Medical University Cancer Hospital between January 2014 and July 2020 were enrolled in this study. The inclusion criteria were as follows: (1) definite pathological diagnosis of “neuroblastoma” or “ganglion cell neuroblastoma” and (2) assignment to the high-risk group based on the Children's Oncology Group (COG) risk classification system 1 and treatment with the CCCG-NB-2015 regimen (Figure 1). The exclusion criteria were as follows: (1) stem cell transplantation after progression or recurrence in the first-line treatment and (2) incomplete data or loss to follow-up. The following characteristics were collected: the demographic characteristics including age and gender; disease characteristics including tumor primary site, INPC classification, bone marrow infiltration status, MYCN status, INSS staging, NSE and LDH levels at diagnosis, imagine defined risk factors, and efficacy evaluation, treatment regime including surgery, radiotherapy, chemotherapy and autologous stem cell transplantation.

### Treatment regimen and response assessment

Children were staged based on the International Neuroblastoma Staging System (INSS), assigned to the high-risk group based on the COG risk classification system and treated by the CCCG-NB-2015 protocol 7. Briefly, the treatment regimen incorporated an induction phase including eight cycles of chemotherapy with 21 days a cycle (or 12 cycles of chemotherapy without SCT) and surgical resection; a consolidation phase including single or tandem cycles of myeloablative therapy and SCT; and a postconsolidation phase including radiation therapy to the primary tumour sites and residual metastatic sites and retinoid therapy (Figure 1, Table S1). The treatment response was assessed based on the International Neuroblastoma Response Criteria (INRC) 7.

### Myeloablative chemotherapy and stem cell transplantation

Autologous peripheral blood stem cell collection was performed after 2-4 cycles of induction chemotherapy. Informed consent was signed before collection, and granulocyte colony stimulating factor was used for stem cell mobilization. Before collection, a central venous catheter was placed to establish venous access, and the collection target was CD34 cells >3×10^6^/kg. If necessary, collection was performed multiple times to obtain sufficient cells, which were frozen at -196 °C on the day of collection. At our centre, harvested stem cells are immunostained with GD2 antibody to ensure that the reinfused stem cells are not affected by the tumor. As shown in Table 1, the first myeloablative chemotherapy consisted of busulfan/melphalan (Bu/Mel), and the second regimen was carboplatin, etoposide, and cyclophosphamide (CEC). The day of engraftment was defined as the first of 3 consecutive days with an absolute neutrophil count (ANC) >500/μL. For common adverse reactions, we also administered preventive measures, such as corticosteroids to prevent interstitial pneumonia, levetiracetam to prevent epilepsy, cotrimoxazole to prevent Pneumocystis carinii, and ursodeoxycholic acid to prevent VOD. For example, levetiracetam is routinely used to prevent busulfan-induced seizures, with diazepam or phenobarbital given during seizures.

### Statistical analysis

The primary outcome was event-free survival (EFS), which was defined as the time (months) from diagnosis to the event (including progression, recurrence, or death from any cause) or the date at the end of follow-up. Overall survival (OS) was defined as the time (months) from diagnosis to death or the end of the follow-up date. The follow-up ended on July 31, 2020. SPSS 25.0 software was used for data analysis, the chi-square test was used for categorical variables for comparisons between groups, and the Mann-Whitney U test was used for continuous variables for comparisons between groups. The Kaplan-Meier method was used to calculate the survival rate and draw survival curves, and the log-rank method was used for statistical analysis. Bilateral *p*<0.05 was considered statistically significant.

## Results

### Characteristics of study patients

The study included a total of 90 children with high-risk neuroblastoma. The median age at diagnosis was 42 months (11-97 months), and the male-to-female ratio was 1.43. A total of 31.1% of children had abnormal amplification of the MYCN gene. After induction chemotherapy, 84 cases (93.3%) were evaluated as CR/VGPR, as shown in Table 2. The median follow-up time was 29 (5-78) months. The 3-year EFS and OS of all 90 eligible patients were 56.8% (95% CI 45.2%-68.4%) and 70.6% (95% CI 59.8%-81.4%), respectively. In our case, all stem cells collected were negative for GD2 staining.

### Analysis of the effect of autologous peripheral blood stem cell transplantation

**Figure 2:**
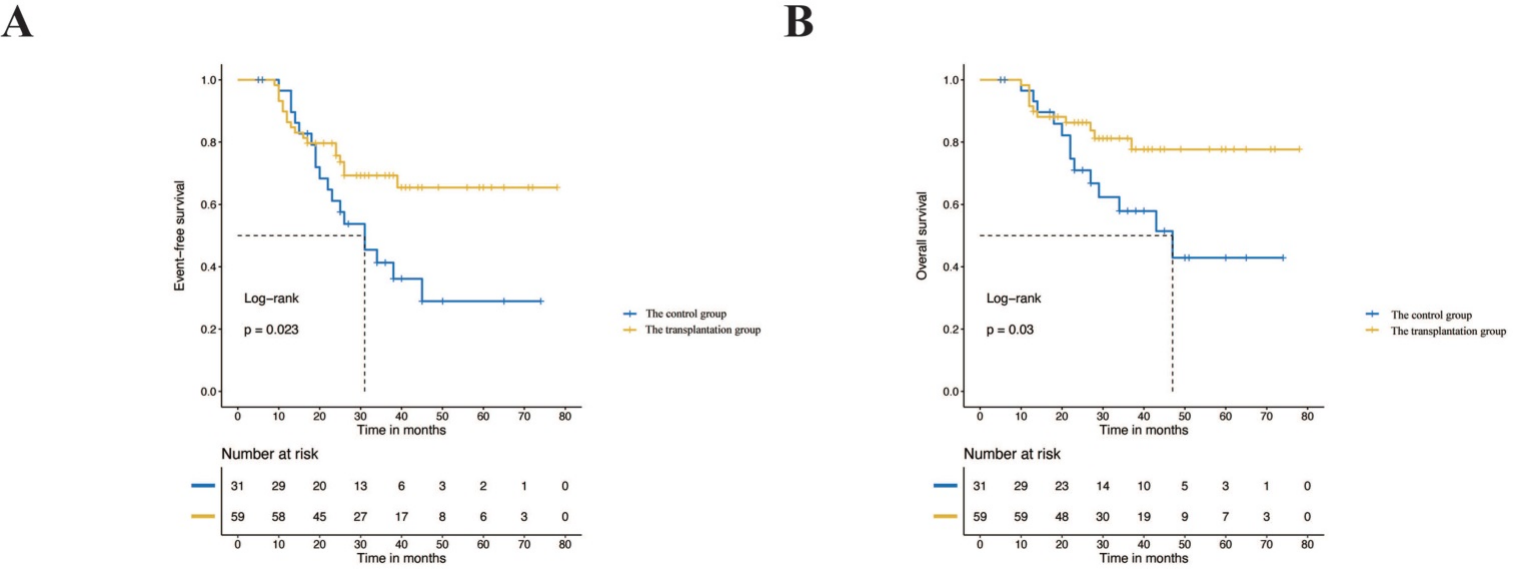
Event-free survival rates and overall survival rates of the transplantation group and control group.

Fifty-nine (65.6%) children received single or tandem autologous stem cell transplantation. The median number of induction chemotherapy cycles in the control group (without SCT) was 12 (6-13), with 8 (6-12) cycles in the transplantation group, which showed a statistically significant difference (Z=-4.186, *p*=0.000). The two groups showed no significant differences in the distribution of baseline characteristics (*p*>0.05). In the transplantation group, 46 children (78.0%) received radiotherapy, while 19 children (61.3%) received radiotherapy in the control group (*p*=0.093). After induction chemotherapy, 96.6% and 87.1% of the transplantation group and the control group achieved CR/VGPR, respectively, without a significant difference (*p*=0.202), as shown in Table 2. In summary, there was no significant difference in the distribution of baseline characteristics and treatment response between the transplantation group and the control group. A total of 58.1% and 30.5% of the control group and the transplant group experienced recurrence, progression or death, respectively. The survival curve showed that the prognosis of the transplantation group was significantly improved compared with that of the control group. The 3-year EFS was 65.5% vs. 41.3% (*p* = 0.023), while the 3-year OS was 77.7% vs. 57.9% (*p*=0.03) (Figure 2).

### Analysis of the effect of tandem and single autologous peripheral blood stem cell transplantation

**Figure 3:**
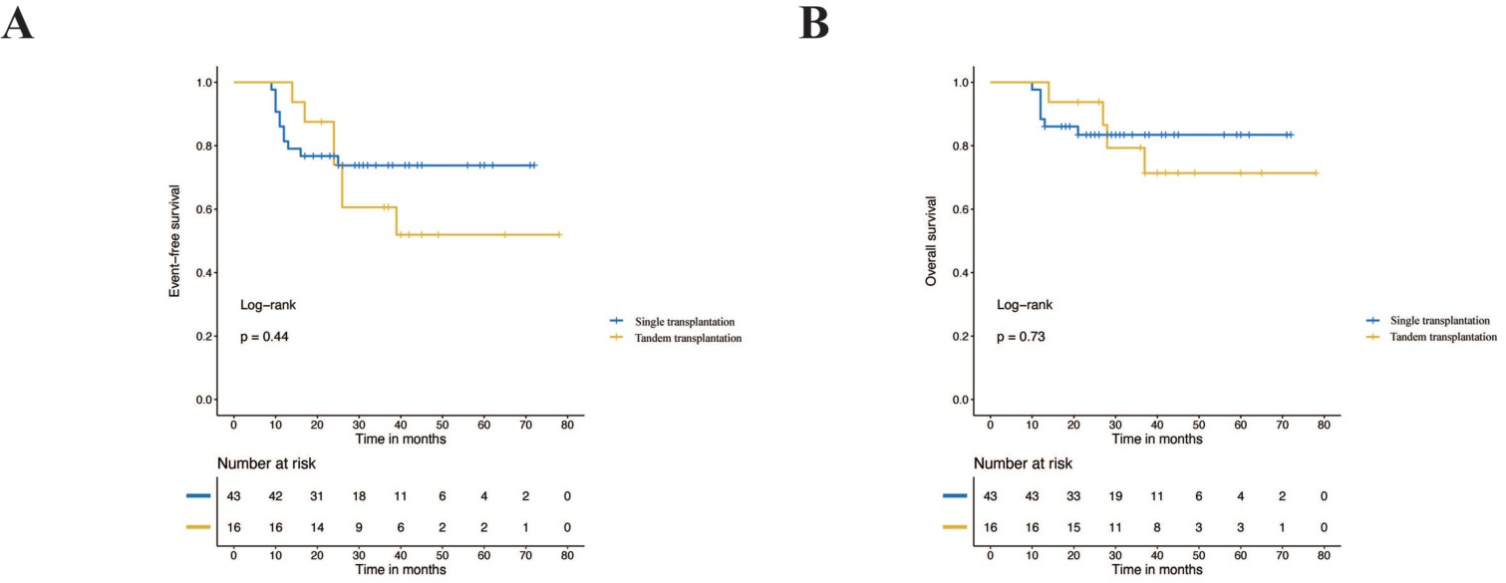
Event-free survival rates and overall survival rates of the single transplantation group and tandem transplantation group.

Among the children in the high-risk group who underwent transplantation, 16 children (27.1%) received tandem stem cell transplantation. There was no significant difference between the single transplantation group and the tandem transplantation group in the baseline characteristics and treatment response (*p*> 0.05) (Table 3). The survival analysis showed that the prognosis of tandem transplantation was not significantly different from that of single transplantation. The 3-year EFS was 51.9% vs. 73.8% (*p*=0.44), and the 3-year OS was 71.4% vs. 83.4% (*p*=0.73) (Figure 3).

### Toxicity

The most common adverse reaction in autologous peripheral blood stem cell transplantation was agranulocytosis with fever, with an overall incidence of 81.4%, of which 79.1% of cases were in the single transplantation group and 87.5% were in the tandem transplantation group. Infections caused by agranulocytosis are more common. Among all patients who received stem cell transplantation, 39.0% developed agranulocytosis-related infections, of which gastrointestinal infections were almost the most common, followed by upper respiratory tract infections. The second most common adverse reaction was gastrointestinal, including nausea and vomiting, diarrhoea, and constipation. The overall incidence was 49.2% and 59.3% in the single and tandem transplantation groups, respectively. Two children had epileptic seizures during myeloablative chemotherapy, and three children had bleeding manifestations (nasal bleeding), which were relieved after symptomatic treatment. Veno-occlusive disease (VOD) or sinusoidal obstruction syndrome (SOS) is a complication requiring special mention characterized that occurs after high-dose chemotherapy and hematopoietic stem cell transplantation (HSCT) with Bu/Mel regimen. In our case, only one child developed hepatomegaly with transient elevation of transaminases and bilirubin. We treated it according to the VOD/SOS treatment method, and the child was eventually cured. Another child also had transient elevation of liver function, and transaminases were reduced to normal after hepatoprotective and choleretic therapy. Nine patients developed grade I or II (according to NYHA classification) cardiac insufficiency, mainly manifested as shortness of breath after exercise and the Left Ventricular Ejection Fractions (LVEF) was slightly lower than before transplantation. But after oxygen inhalation and the circulation improving, the patients' symptoms improved significantly. The overall patient tolerance was acceptable, and there were no serious grade 3 or 4 adverse reactions. In our cases, all chemotherapy was performed according to the CCCG-2015 NB protocol. The main side effects of chemotherapy during the period were bone marrow suppression and gastrointestinal reactions. The severe and persistent nephrotoxicity, cardiotoxicity, or hearing impairment due to chemotherapy drugs did not occur in our cases, but long-term follow-up was needed. Severe bone marrow suppression, nausea and vomiting can be recovered after corresponding symptomatic treatment. Neutrophil engraftment was reached after a median of 11 days (range, 8 to 18 days) for single transplantation and 10 days (range, 8 to 20 days) for tandem transplantation, and the difference was not statistically significant (*p* =0.249) (Table 4).

### Analysis of the effect of radiotherapy on prognosis in transplant patients

**Figure 4:**
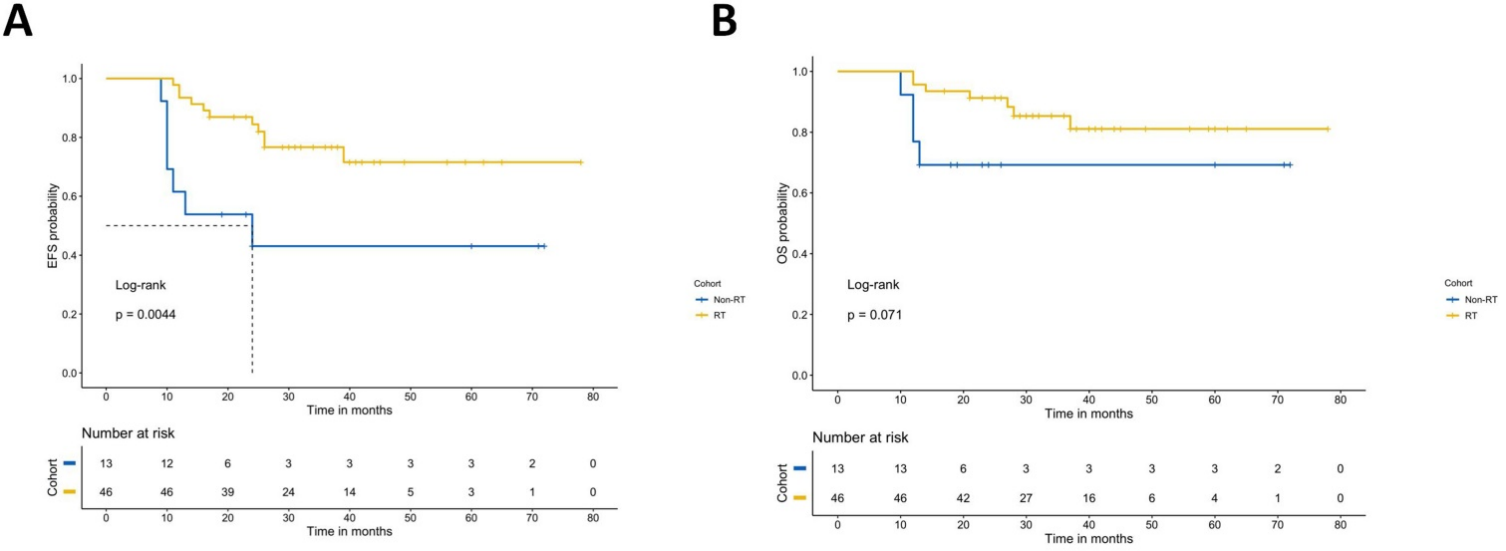
Event-free survival rates and overall survival rates of the radiotherapy group and nonradiotherapy group.

Among the children in the high-risk group who underwent transplantation, 46 children (76.1%) received radiotherapy. The results show that radiotherapy after transplantation can improve the EFS of NB. The survival analysis showed that the prognosis of radiotherapy was significantly different from that of nonradiotherapy. The 3-year EFS was 76.7% vs. 43.1% (*p*=0.004), and the 3-year OS was 81.1% vs. 69.2% (*p*=0.07) (Figure 4).

## Discussion

Neuroblastoma is the most common extracranial solid tumour in children. Although the prognosis continues to improve, the long-term survival of the high-risk group is still poor, and autologous peripheral blood stem cell transplantation has become the standard therapy for the high-risk group in the consolidation phase. This study retrospectively analyzed the efficacy of autologous peripheral blood stem cell transplantation for high-risk neuroblastoma in our centre, and showed that stem cell transplantation with the CCCG-2015 protocol can significantly improve the prognosis of children in the high-risk group, with manageable toxicity. However, the efficacy of tandem transplantation still needs further research and verification.

In China, high-risk neuroblastoma was rarely treated with stem cell transplantation after induction chemotherapy before 2009 and was characterized by a poor prognosis. Subsequently, the CCCG formulated the CCCG-NB-2009 treatment regimen as a consensus on the diagnosis and treatment of neuroblastoma and incorporated SCT into the treatment of high-risk neuroblastoma. Based on this treatment regimen, the prognosis of high-risk neuroblastoma in our centre improved greatly, with a 3-year progression-free survival rate increasing from 18.2% to 51.5%. Subsequently, CCCG further improved the treatment regimen, optimized induction chemotherapy, introduced tandem stem cell transplantation, and formed the CCCG-NB 2015 regimen. However, in a developing country such as China, stem cell transplantation is a huge financial burden for most families, which is why some patients choose to undergo stem cell transplantation, and some do not. All transplant and nontransplant groups were relatively randomized in our centre.

### Myeloablative chemotherapy regimen

There are many regimens for myeloablative chemotherapy for autologous peripheral blood stem cell transplantation, and the application of melphalan, thiotepa and other drugs has made a great progress in the treatment of high-risk neuroblastoma 8-14. Previous studies have shown that busulfan and melphalan improve the prognosis of high-risk neuroblastoma with an adequate response to induction treatment, cause fewer severe adverse events than CEM and should be considered standard high-dose chemotherapy for single transplantation 6. Therefore, our centre applied this regimen, which yielded an encouraging prognosis with acceptable toxicity. The myeloablative chemotherapy regimen of tandem transplantation has not yet been unified. The chemotherapy regimen of tandem transplantation recommended by COG is thiotepa/cyclophosphamide followed by carboplatin/etoposide/melphalan, with a 3-year EFS of 61.6% 4. Limited by the availability and expensive expenditure of thiotepa in China, carboplatin, etoposide and cyclophosphamide (CEC) from the POG 9342 trial became the chemotherapy regimen for the second transplantation, with a 5-year EFS of 58.3% reported by previous research 15, 16. Therefore, in our centre, the myeloablative chemotherapy regimen for tandem transplantation is Bu/Mel followed by CEC regimen. Sixteen children received tandem transplantation in this study and showed a favourable prognosis with a 3-year EFS of 51.9%.

### Stem cell transplantation significantly improves prognosis

Autologous peripheral blood stem cell transplantation can significantly improve the prognosis of high-risk neuroblastoma. A series of large international randomized controlled trials have shown that autologous peripheral blood stem cell transplantation can significantly improve the prognosis of high-risk neuroblastoma, with a 3-year EFS increase to 38%-50% 4, 10, 17, 18. Our results revealed that the 3-year EFS of the high-risk group with stem cell transplantation reached 65.5%, which was slightly higher than that reported in the literature. More than 90% of the transplantation group achieved CR/VGPR after induction chemotherapy, which was higher than the 40%-70% reported in the literature and may be responsible for the better prognosis 4, 6, 9. As shown in previous studies, the treatment response after induction is also an important prognostic factor for high-risk neuroblastoma 4, 19. However, some medical centres in China have a conservative view of the prognostic value of stem cell transplantation. This may be because some researchers believe that stem cell transplantation can improve EFS but is useless for OS 20. In addition, the high cost and parents' anxiety about adverse reactions also hinder the clinical application of stem cell transplantation to a certain extent 21. The proportion of high-risk neuroblastoma patients receiving stem cell transplantation in our centre was only 65.6%, which also showed the limitations of stem cell transplantation in actual clinical application. In summary, autologous peripheral blood stem cell transplantation can significantly improve the prognosis of high-risk neuroblastoma; however, it also poses certain difficulties in actual clinical application.

### Effectiveness of tandem transplantation

The efficacy of tandem transplantation is still under research. The efficacy and feasibility of tandem transplantation have been reported frequently in high-risk neuroblastoma during the past decade 14, 22, 23. A randomized multicentre clinical trial on single and tandem autologous stem cell transplantation conducted by COG profoundly influenced the subsequent treatment strategy. In this trial, patients in the single transplantation group received the conditioning regimen CEM, while cyclophosphamide/thiotepa and a dose-reduced CEM conditioning regimen were used for the tandem transplantation group. The results showed that tandem transplantation significantly improved the prognosis compared with single transplantation in high-risk neuroblastoma, with the 3-year EFS increasing from 48.4% to 61.6% (P=0.006). Although conditioning regimens vary greatly in different studies, tandem transplantation has gradually become an indispensable part of high-risk neuroblastoma treatment regimens 22,24. Many studies have shown that tandem transplantation can improve the prognosis of high-risk neuroblastoma 14, 24. The randomized clinical trial of ANBL0532 conducted by COG showed that, compared with single transplantation, tandem transplantation increased the 3-year EFS of high-risk neuroblastoma from 48.4% to 61.6% (*p*=0.006), without improvement in OS (*p*=0.25) 4. Tandem transplants clearly showed a trend for better EFS, although the difference was not statistically significant in our cohort. The following reasons may be responsible for this result. First, only sixteen children received tandem transplantation, which was limited by the design of the retrospective study. Second, patients in our study showed a better response to the induction phase with extended induction chemotherapy than patients in a previous study did 4. The ability of tandem transplantation to further improve the prognosis may not be clear. Third, different treatment regimens may be important factors limited by the availability of immunotherapy, which was not included in the treatment regimen of this study, while 70.4% of patients received immunotherapy in the previous study 4, 25. Last, the Bu/Mel regimen itself has been proven to be the best choice in a single transplant situation, after the first transplantation with the Bu/Mel regimen, the second transplantation may be less effective than stated in the ANBL0532 report. Therefore, further research is needed to determine whether a second transplantation is needed and how to choose the second transplantation program after Bu/Mel transplantation. In summary, tandem stem cell transplantation still needs further research and verification.

### Toxicity

The safety of autologous peripheral blood stem cell transplantation has also been extensively verified 4, 6. This study showed that the acute toxicity of stem cell transplantation mainly included agranulocytosis with fever and gastrointestinal reactions. There was no significant difference in the incidence of toxicities between single transplantation and tandem transplantation, which is similar to the findings of previous studies 6, 22. After symptomatic treatment, the above symptoms can all be relieved. Although our enrolled cases did not develop definite VOD/SOS or severe heart failure, this does not mean that such side effects will not occur. It is true that children who receive multiple cycles of chemotherapy and then undergo stem cell transplantation have a greatly increased chance of adverse reactions such as VOD/SOS, heart failure, TMA, and renal failure. The challenge with Bu/Mel regimen has been the toxicity and though the data in our study does not reveal much of toxicity it should be acknowledged. We should pay more attention to these in the future, especially after multiple cycles of induction chemotherapy. For possible toxic reactions, we give preventive treatment in advance, which can reduce the occurrence of these complications. The median neutrophil engraftment times of single and tandem transplantation in this study were 11 days and 10 days, respectively, which is consistent with literature reports 12, 26. In summary, autologous peripheral blood stem cell transplantation is safe and tolerable.

### Can extended chemotherapy replace stem cell transplantation?

Extending induction chemotherapy is not a substitute for stem cell transplantation. The current induction chemotherapy is approximately 6-8 cycles 27. However, this study was based on the CCCG-NB-2015 regimen, and the median number of cycles of induction chemotherapy was eight in the transplantation group and twelve in the control group. Despite extension of the chemotherapy dose, the prognosis of the control group was still lower than that of the transplantation group, and the 3-year EFS was 41.3% and 65.5%, respectively (*p*=0.023). Similar to previous studies, stem cell transplantation had a better prognosis than an additional cycle of maintenance chemotherapy after induction chemotherapy, and the 3-year EFS was 47% and 31% (*p*=0.02), respectively 28. Even after acceptance of an additional three cycles of intensive chemotherapy after induction therapy, the prognosis was still inferior to stem cell transplantation, and the 5-year EFS was 19% and 30%, respectively (*p*=0.04) 23. We also confirmed that, compared with stem cell transplantation, increasing the dose of induction chemotherapy cycles does not improve the prognosis. Therefore, induction chemotherapy is not an alternative for high-risk neuroblastoma without transplantation contraindications. Parents should still be persuaded to accept stem cell transplantation as much as possible.

### Effectiveness of radiotherapy after transplantation

In the consolidation therapy phase of high risk neuroblastoma, radiation to the primary tumor site is indicated after myeloablative therapy 29. Our data demonstrates that continued radiotherapy after transplantation can significantly improve the prognosis of neuroblastoma. In China, many patients give up radiotherapy because they are worried about the side effects. In our follow-up of these children, the short-term adverse reactions of radiotherapy were mainly bone marrow suppression, nausea and vomiting, that no serious organ damage was observed. We did not observe obvious abnormal growth and development, and the routine follow-up of patients does not reflect the existence of short stature, scoliosis, hearing impairment or other phenomena. Therefore, longer follow-up with more focus on growth and development is needed to study the long-term toxicity of radiotherapy to children.

### Limitations

Limited by the single-centre, retrospective design, a small number of patients were enrolled in this study. In addition, it is still necessary to extend the follow-up time to evaluate the long-term prognostic value of stem cell transplantation. GD2 antibody drug will not be available in China until 2021, so the patients in our study did not receive immunotherapy, which is indeed one of the main treatments for improving neuroblastoma EFS.

## Supplementary Material

Supplementary table.

## Figures and Tables

**Table 1:** Myeloablative chemotherapy of autologous peripheral blood stem cell transplantation for high-risk neuroblastoma

Regimen	Autologous peripheral blood stem cell transplantation time (days)								
	-8	-7	-6	-5	-4	-3	-2	-1	0
**Single transplantation group**									PBSC infusion
Busulfan^a^	√	√	√	√					
Melphalan^b^						√			
**Tandem transplantation group**									
#1									PBSC infusion
Busulfan^a^	√	√	√	√					
Melphalan^b^						√			
#2									PBSC infusion
CBP^c^	√	√	√						
VP16^d^	√	√	√						
CTX^e^				√	√				

a: Busulfan: 1 mg/kg/dose q6h, b: Melphalan: 140 mg/m^2^; c: CBP: 600 mg/m^2^, d: VP16: 500 mg/m^2^, e: CTX: 1800 mg/m^2^.

**Table 2:** Characteristics of 90 children with high-risk neuroblastoma

Name	Control group (N/%)	Transplantation group (N/%)	*P* value
**Age (month)**			1.000
<18	1 (3.2)	3 (5.1)	
≥18	30 (96.8)	56 (94.9)	
**Primary site**			0.519
Retroperitoneum	25 (80.6)	52 (88.1)	
Non-retroperitoneal	6 (19.4)	7 (11.9)	
**INPC**			0.084
FH	14 (45.2)	16 (27.1)	
UH	17 (54.8)	43 (72.9)	
**Bone marrow involvement**			0.903
Yes	24 (77.4)	45 (76.3)	
No	7 (22.6)	14 (23.7)	
**MYCN amplification status**			0.431
No	23 (74.2)	39 (66.1)	
Yes	8 (25.8)	20 (33.9)	
**INSS stage**			0.969
1, 2, 3, 4s	4 (12.9)	6 (10.2)	
4	27 (87.1)	53 (89.8)	
**NSE**			0.124
<200 ng/ml	9 (29.0)	27 (45.8)	
≥200 ng/ml	22 (71.0)	32 (54.2)	
**LDH**			1.000
<1400 U/L	27(87.1)	50 (84.7)	
≥1400 U/L	4 (12.9)	9 (15.3)	
**IDRFs**			0.090
No	4 (12.9)	17 (28.8)	
Any	27 (87.1)	42 (71.2)	
**Radiation therapy**			0.093
No	12 (38.7)	13 (22.0)	
Yes	19 (61.3)	46 (78.0)	
**Efficacy evaluation**			0.202
CR/VGPR	27 (87.1)	57 (96.6)	
PR	4 (12.9)	2 (3.4)	

INPC: International Neuroblastoma Pathology Classification. FH: Favorable Histology. UH: Unfavorable Histology; INSS: International Neuroblastoma Staging System; NSE: Neuron-specific enolase; LDH: lactate dehydrogenase; IDRFs: Image-defined Risk Factors; Efficacy evaluation: efficacy evaluation after induction chemotherapy. CR: complete remission. VGPR: very good partial remission. PR: partial remission.

**Table 3:** Characteristics of 59 cases of autologous peripheral blood stem cell transplantation

Name	Single (N/%)	Tandem (N/%)	*P* value
**Age (month)**			0.556
<18	3 (7.0)	0 (0.0)	
≥18	40 (93.0)	16 (100.0)	
**Primary site**			1.000
Retroperitoneum	38 (88.4)	14 (87.5)	
Non-retroperitoneal	5 (11.6)	2 (12.5)	
**INPC**			0.581
FH	13 (30.2)	3 (18.8)	
UH	30 (69.8)	13 (81.2)	
**Bone marrow involvement**			0.838
Yes	32 (74.4)	13 (81.2)	
No	11 (25.6)	3 (18.8)	
**MYCN amplification status**			0.134
No	26 (60.5)	13 (81.2)	
Yes	17 (39.5)	3 (18.8)	
**INSS stage**			0.902
1, 2, 3, 4s	5 (11.6)	1 (6.3)	
4	38 (88.4)	15 (93.7)	
**NSE**			0.437
<200 ng/ml	21 (48.8)	6 (37.5)	
≥200 ng/ml	22 (51.2)	10 (62.5)	
**LDH**			1.000
<1400 U/L	36 (83.7)	14 (87.5)	
≥1400 U/L	7 (16.3)	2 (12.5)	
**IDRFs**			0.473
No	14 (32.6)	3 (18.8)	
Any	29 (67.4)	13 (81.2)	
**Radiation therapy**			0.152
No	12 (27.9)	1 (6.3)	
Yes	31 (72.1)	15 (93.7)	
**Efficacy evaluation**			0.472
CR/VGPR	42 (97.7)	15 (93.7)	
PR	1 (2.3)	1 (6.3)	

INPC: International Neuroblastoma Pathology Classification. FH: Favorable Histology. UH: Unfavorable Histology; INSS: International Neuroblastoma Staging System; NSE: Neuron-specific enolase; LDH: lactate dehydrogenase; IDRFs: Image-defined Risk Factors; Efficacy evaluation: efficacy evaluation after induction chemotherapy. CR: complete remission. VGPR: very good partial remission. PR: partial remission.

**Table 4:** Adverse reaction in autologous peripheral blood stem cell transplantation

Complications	Single (N/%)	Tandem (N/%)	*p*
Fever	34 (79.1)	14 (87.5)	0.716
Nausea and vomiting	20 (46.5)	9 (56.2)	0.506
Diarrhoea and constipation	24 (55.8)	11 (68.8)	0.369
Mucositis	6 (14.0)	1 (6.3)	0.718
Heart failure	9 (20.9)	4 (25)	1.000
Epileptic seizures	2 (4.7)	1 (6.3)	1.000
Nasal bleeding	3 (7.0)	1 (6.3)	1.000
Neutrophil engraftment time (day)	11 (8-18)	10 (8-20)	0.249

## Abbreviations

NB: Neuroblastoma
CCCG: the Chinese Children's Cancer Group
COG: the Children's Oncology Group
INSS: the International Neuroblastoma Staging System
INRC: the International Neuroblastoma Response Criteria
SCT: Stem Cell Transplantation
Bu/Mel: Bulsufan/Melphalan
CEC: Carboplatin, Etoposide, and Cyclophosphamide
EFS: Event-Free Survival
OS: Overall Survival
CEM: Carboplatin, Etoposide, and Melphalan
MM: Bulsufan/Melphalan
INPC: International Neuroblastoma Pathology Classification
FH: Favorable Histology
UH: Unfavorable Histology
NSE: Neuron-Specific Enolase
LDH: Lactate Dehydrogenase
IDRFs: Image-defined Risk Factors
CR: Complete Remission
VGPR: Very Good Partial Remission
PR: Partial Remission
