# Supplementary material for: Analysis of the efficacy of autologous peripheral blood stem cell transplantation in high-risk neuroblastoma
Source: Int J Med Sci. 2022 Sep 25;19(11):1715–23. doi: 10.7150/ijms.76305 (PMC9553861; doi:10.7150/ijms.76305)
Supplement: Supplementary file 1 — Supplementary table. [file ijmsv19p1715s1.pdf]

Table S1：Chemotherapy regimen for high-risk group neuroblastoma（ CCCG-NB 2015 ）

Course	Scheme
1	CTX*+TOPO
2	CTX*+TOPO
	Response evaluation
3	CDDP+VP-16
4	CTX+DOXO+VCR+MESNA
	Response evaluation
	Autologous peripheral blood stem cell collection
	Operation
5	CDDP+VP-16
6	CTX+DOXO+VCR+MESNA
	Response evaluation
7	CTX*+TOPO
8	CDDP+VP-16
	Response evaluation
undergo HSCT	Not undergo HSCT
ABMT1	CTX+DOXO+VCR+MESNA
Radiotherapy	CTX*+TOPO
ABMT2	CDDP+VP-16
	CTX+DOXO+VCR+MESNA
	Radiotherapy
	Response evaluation
13-cis-RA 160mg/m2，14day/month，total of 6 months	

Cyclophosphamide(CTX)*：400 mg/m2. d1-d5(<12 kg: 13.3 mg/kg)
Topotecan(TOPO)：1.2 mg/m2. d1-5.(Replaced by Irinotecan，120mg/m2. d1-3)
Cisplatin(CDDP)：50 mg/m2. d1-4 (< 12kg: 1.66 mg/kg)
Etoposide(VP-16)：200 mg/m2. d1-3 (<12 kg: 6.67 mg/kg)
Cyclophosphamide(CTX)：1800 mg/m2.d1-2 (< 12 kg: 60mg/kg)
Mesna：420 mg/m2. d1-2 q4hx3
Epirubicin(DOXO)：25 mg/m2. d1-3 (< 12 kg: 0.83mg/kg)
Vincristine(VCR)： < 12 mon: 0.017mg/kg d1-3
> 12 mon and> 12 kg: 0.67 mg/m2 d1-3
>12 mon and< 12 kg: 0.022 mg/kg d1-3
